# Two‐decade battle with myasthenia gravis: A breakthrough case report on the long‐term success with eculizumab and ravulizumab treatment

**DOI:** 10.1002/ccr3.9547

**Published:** 2024-11-04

**Authors:** Stefan Quasthoff

**Affiliations:** ^1^ Department of Neurology Medical University of Graz Graz Austria

**Keywords:** eculizumab, generalized myasthenia gravis, myasthenia gravis, ravulizumab

## Abstract

This unique case of generalized myasthenia gravis shows sustained stability of a patient's condition for 3 years with eculizumab/ravulizumab treatment following 16 years of refractory disease. It highlights the long‐term effectiveness of C5 inhibitors in a real‐world setting, aiding physicians in their decision‐making for refractory cases and treatment discontinuation scenarios.

## 1 INTRODUCTION

Myasthenia gravis (MG) is a rare, chronic autoimmune disorder affecting the neuromuscular junction (NMJ).^1^ MG is characterized by muscle fatigability and weakness, initially only affecting the eye muscle in most cases.^2^ However, MG often progresses to generalized MG (gMG), affecting the muscles of the head, neck, trunk, limbs, and/or respiratory system. This progression occurs in about 80% of patients within 2–3 years of disease onset.^2^ Additionally, approximately 80%–90% of patients with gMG have auto‐antibodies targeting the acetylcholine receptor (AChR), with complement activation playing a crucial role in the pathologic mechanism in patients who are positive for anti‐AChR antibodies (AChR^+^).^2^, ^3^ Several subtypes of MG exist based on clinical presentation, auto‐antibody profile, and pathology of the thymus, including early‐onset, late‐onset, and thymoma‐associated‐MG.^4^ While early‐onset MG typically occurs before the age of 40 and is more common in female patients, late‐onset MG is more common in males over the age of 60 without thymoma.^4^

Myasthenic crisis, a common complication of MG affecting 15%–20% of patients, is characterized by respiratory failure requiring mechanical ventilation and intensive care.^2^, ^5^ Myasthenic crises often lead to substantial long‐term physical and mental consequences.^2^, ^5^ The burden of MG is considerable, negatively impacting patients' daily activities; quality of life (QoL); and emotional, social, and economic well‐being.^5^

The current MG treatment algorithm, particularly long‐term use of corticosteroids or nonsteroidal/nonspecific immunosuppressive therapies, has serious, often troublesome, side effects and unpredictable treatment responses.^1^, ^5^ This leads to patient dissatisfaction and reduced treatment adherence, further complicating MG management.^1^, ^5^ Targeted approaches to gMG management, namely eculizumab (Soliris®) and ravulizumab (Ultomiris®), were approved in the United States and Europe in 2017 and 2022,^6^, ^7^ respectively. Data from two open‐label extension studies indicate the long‐term safety and sustained effectiveness of both medicines.^2^, ^8^

Eculizumab, the first complement‐targeting drug approved for complement‐mediated diseases,^3^ is a humanized monoclonal antibody that binds specifically and with high affinity to the human terminal complement protein C5.^5^ This prevents destruction of the NMJ and consequent muscle weakness and fatigability.^5^ Ravulizumab, also a C5 inhibitor, is engineered from eculizumab to have a longer half‐life, maintaining therapeutic serum concentrations over an 8‐week dosing interval, compared to a 2‐week interval with eculizumab.^7^

Despite the clinical benefits of these targeted treatments, the success of their use in real‐world disease management is multifactorial. Confirmation of their long‐term effectiveness and safety in routine clinical practice is needed. Additionally, strategies for overcoming challenges associated with their accessibility, such as high cost and limited availability, are necessary.^9^ We report a patient with 20 years of AChR^+^ gMG, who after 16 years of refractory disease achieved and, importantly, has maintained a stable condition for the last 3 years with eculizumab/ravulizumab therapy.

## 2 CASE HISTORY/EXAMINATION

**FIGURE 1:**
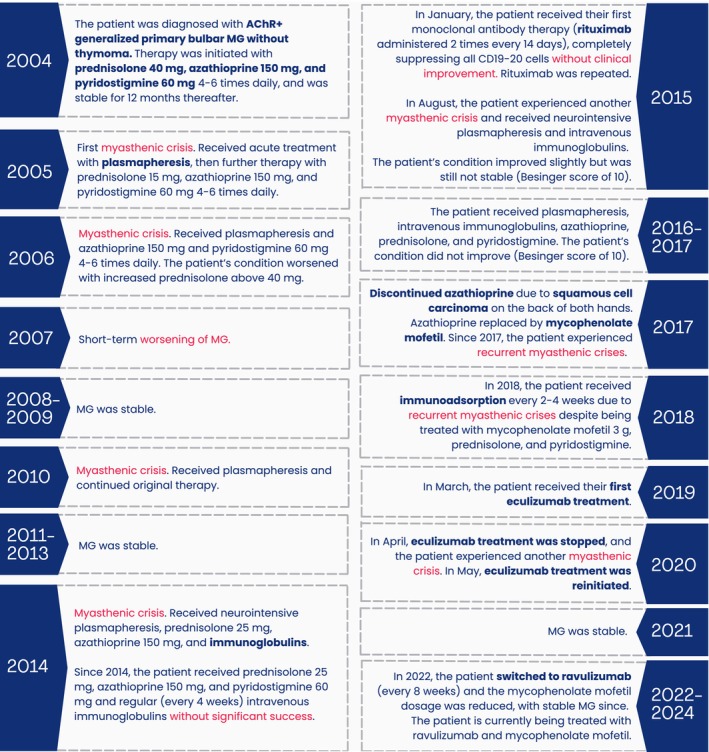
Patient's medical history including timeline of events and course of illness and treatments. AChR+, acetylcholine receptor positive; CD19‐20, Cluster of Differentiation 19 and 20; MG, Myasthenia Gravis.

**FIGURE 2:**
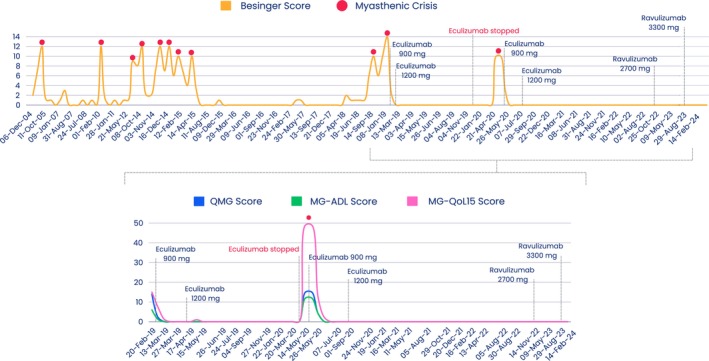
Patient's medical history including myasthenic crises (red circles), eculizumab and ravulizumab treatment regimens, and related patient‐ and physician‐reported outcome measure scores assessing myasthenia gravis symptoms and severity, quality of life, and activities of daily living. MG‐ADL, Myasthenia Gravis Activities of Daily Living; MG‐QoL15, Myasthenia Gravis Quality of Life 15‐item questionnaire; QMG, Quantitative Myasthenia Gravis.

A detailed description of the patient's history, including treatments and outcomes, are presented in Figures 1 and 2.

Briefly, in 2004, a 77‐year‐old male was diagnosed with AChR^+^ gMG with primary bulbar symptoms without thymoma. Initial treatment included prednisolone, azathioprine, and pyridostigmine. Over the years, the patient experienced multiple myasthenic crises, exacerbated by infections and stress, and underwent various treatments with limited success. In 2017, azathioprine was replaced by mycophenolate due to the development of carcinoma. However, the patient's condition remained unfavorable until initiating eculizumab in 2019.

## 3 METHODS

At diagnosis, an AChR antibody level of 19.4 pg/mL was noted, and a computed tomography (CT) scan revealed no evidence of thymoma or thymic hyperplasia. Therapy was initiated with prednisolone 40 mg, azathioprine 150 mg, and pyridostigmine 60 mg 4–6 times daily. The patient was stable for 12 months thereafter before experiencing his first myasthenic crisis. This first myasthenic crisis was acutely treated with plasmapheresis, followed by more prednisolone, azathioprine, and pyridostigmine. Over the next 10 years, the patient experienced several more myasthenic crises, exacerbated by infections (such as influenza) and physical and psychological stressors. Myasthenic crises were treated similarly with steroids and non‐steroidal immunosuppressants (e.g., azathioprine and mycophenolate mofetil) at variable doses. However, these treatments had varying responses, mostly with minimal success and in some cases worsening of MG. In 2015, the patient received his first monoclonal antibody therapy (rituximab) without clinical improvement. Rituximab was administered again, but without effect. In 2017, the patient discontinued azathioprine due to squamous cell carcinoma on the back of both hands, and azathioprine was replaced by mycophenolate mofetil (a different immunosuppressant). The squamous cell carcinoma was treated with surgery and photochemotherapy (psoralen plus ultraviolet A). Despite a rigorous treatment regimen, the patient continued to experience recurring myasthenic crises, prompting immunoadsorption every 2–4 weeks, with limited success.

The patient first received eculizumab in 2019 (loading dose of 900 mg, followed by a maintenance dose of 1200 mg). In April of 2020, the patient had to discontinue eculizumab treatment for a short period because his insurance provider no longer covered the cost. In 2022, the patient switched from eculizumab to ravulizumab (loading dose of 2700 mg, followed by a maintenance dose of 3300 mg every 8 weeks) because the equivalent dose was more cost effective and the longer intervals between administrations made traveling the long distance to the treatment facility less troublesome.

## 4 CONCLUSION AND RESULTS

Eculizumab significantly improved and stabilized the patient's condition as assessed by several pertinent physician‐ and patient‐reported outcome measures of MG symptoms and severity and QoL (Besinger, quantitative MG [QMG], MG activities of daily living [MG‐ADL], and MG‐QoL [15‐item questionnaire] [MG‐QoL15] scores) (Figures 1 and 2). Discontinuation of eculizumab resulted in the patient experiencing MG regression, including a myasthenic crisis. However, the patient's condition quickly improved upon reinitiating (within 12 days) eculizumab. The switch to ravulizumab occurred without any complications and the patient is currently still being treated every 2 months with ravulizumab and reduced steroid intake. Regular CT scans (every 4 years or in the event of a myasthenic crisis) ruled out thymoma or the need for surgical intervention. In August 2023, the AChR antibody level was 73.5 pg/mL, but with no correlation with the patient's clinical status observed.

## 5 DISCUSSION

Case reports highlighting eculizumab and ravulizumab as effective rescue therapy for MG are increasing, with emerging evidence supporting their positive impact on the disease's long‐term progression.^10^ The current case report confirms the long‐term effectiveness and safety of eculizumab and ravulizumab as a rescue treatment for gMG in a patient who was unresponsive to conventional immunosuppressive therapies for 16 years. For this patient, eculizumab/ravulizumab treatment was a “game changer” in gMG management, not only because of a rapid improvement in clinical outcomes but also the fact that these targeted treatments have maintained efficacy over the last 3 years, with a significant positive impact on the patient's QoL.

Although eculizumab was highly effective in this case of refractory gMG, the patient was required to discontinue its use for a short duration due to the insurance provider's decision to cease funding for the treatment. Similar to a previous report,^11^ discontinuing eculizumab in the current case resulted in significant worsening of the patient's condition. Favorably, the patient's condition quickly improved upon reinitiating eculizumab treatment, on par with the previous report.^11^ After 2 years of eculizumab, the patient was successfully switched to ravulizumab, which was more accessible both logistically and financially. This switch contributed to an improved quality of life by reducing the frequency of trips to the treatment facility, located approximately 2 h away, and lowering the cost for an equivalent dose of ravulizumab. Favorably, the switch occurred without any negative effect on clinical outcomes, in line with results from a recent study, where switching from eculizumab to ravulizumab was found to be effective for patients with severe refractory AChR^+^ gMG in a clinical setting.^12^ Furthermore, it is particularly noteworthy that the patient exhibited such a positive response to treatment even after a prolonged disease duration, despite recent data suggesting that therapeutic outcomes tend to be more favorable when treatment is initiated shortly after diagnosis.^13^ The patient has been receiving ravulizumab treatment since the switch in 2022 and is doing well.

The cost of managing a long‐term disease like gMG varies according to disease progression.^14^ Nevertheless, the economic burden of gMG can be substantial, calculated at roughly 4× more per month on average (USD 5500) in the United States when compared to patients without gMG.^14^ This cost was estimated to be even higher (3× more) among patients with common comorbidities and MG exacerbation or crisis.^14^ With cost and convenience as barriers to the use of anti‐complement agents,^15^, ^16^, ^17^ the current case underscores the urgent need to develop strategies that enhance the accessibility and long‐term use of targeted immunotherapy in life‐long conditions such as gMG.

As real‐world evidence accumulates, the importance of complement C5 inhibitors in gMG treatment is becoming increasingly clear, with eculizumab and ravulizumab offering promising long‐term management for patients with severe clinical conditions and refractory disease. Furthermore, unlike eculizumab, which is primarily reserved for refractory cases, ravulizumab may be considered for earlier intervention in the disease course, potentially even before the manifestation of severe clinical symptoms.

## AUTHOR CONTRIBUTIONS

**Stefan Quasthoff:** Conceptualization; data curation; formal analysis; investigation; methodology; project administration; resources; supervision; validation; visualization; writing – original draft; writing – review and editing.

## FUNDING INFORMATION

This research has received no external funding. Writing assistance was funded by Alexion Pharmaceuticals.

## CONFLICT OF INTEREST STATEMENT

Stefan Quasthoff has received funding for research and consulting from Argenx, Alexion, Astra Zeneca, Biogen, CLS Behring, and Kedrion.

## ETHICS STATEMENT

None.

## CONSENT

Written informed consent was obtained from the patient to publish this report in accordance with the journal's patient consent policy.

## Data Availability

The data supporting this study's findings are available from the corresponding author upon reasonable request.
